# HaploShare: identification of extended haplotypes shared by cases and evaluation against controls

**DOI:** 10.1186/s13059-015-0662-9

**Published:** 2015-05-09

**Authors:** Dingge Ying, Pak Chung Sham, David Keith Smith, Lu Zhang, Yu Lung Lau, Wanling Yang

**Affiliations:** Department of Paediatrics and Adolescent Medicine, LKS Faculty of Medicine, The University of Hong Kong, 21 Sassoon Rd., Pokfulam, Hong Kong; Department of Psychiatry, LKS Faculty of Medicine, The University of Hong Kong, Pokfulam, Hong Kong; Centre for Genomic Sciences, LKS Faculty of Medicine, The University of Hong Kong, Pokfulam, Hong Kong; State Key Laboratory for Emerging Infectious Diseases, The University of Hong Kong, Pokfulam, Hong Kong

## Abstract

Recent founder mutations may play important roles in complex diseases and Mendelian disorders. Detecting shared haplotypes that are identical by descent (IBD) could facilitate discovery of these mutations. Several programs address this, but are usually limited to detecting pair-wise shared haplotypes and not providing a comparison of cases and controls. We present a novel algorithm and software package, HaploShare, which detects extended haplotypes that are shared by multiple individuals, and allows comparisons between cases and controls. Testing on simulated and real cases demonstrated significant improvements in detection power and reduction of false positive rate by HaploShare relative to other programs.

**Electronic supplementary material:**

The online version of this article (doi:10.1186/s13059-015-0662-9) contains supplementary material, which is available to authorized users.

## Background

Although genome-wide association studies (GWAS) have revealed numerous common susceptibility variants for complex diseases [1,2], only a small fraction of disease heritability can be explained [3,4]. Rare variants may be a major source of genetic variations that predisposes individuals to disease [5-7], but their detection is often difficult. The low penetrance or effect size of rare variants, as well as genetic heterogeneity, often means that they are unlikely to be detected by classical linkage analysis, and their low allele frequency significantly reduces the power of association studies. Next generation sequencing technologies can detect rare variants [7], but firmly establishing a link between them and an underlying disease is difficult without robust statistical methods or functional characterization [6,8].

While common variants are mostly shared across ethnic groups, rare variants are more likely to be recent in history and population-specific. Some are recent founder mutations, shared by a number of individuals whose relationship may not be socially known. Recent founder mutations playing a role in a disease should aggregate more in cases than in controls and the haplotypes in which they reside should have been affected by only a limited number of recombination events, unlike haplotypes in the general population. Making use of this distinction may aid the detection of recent haplotypes among cases and facilitate detection of founder mutations.

Many efforts have been made in recent years to identify haplotypes shared IBD, with the aim of locating disease genes [9-13]. A drawback of most of these algorithms is that haplotype frequency was not taken into consideration, which limits the detection power and thus, they are only suitable to detect very long haplotypes shared IBD. PLINK [14] and GERMLINE [15] are two programs that are quite efficient in processing genome-wide SNP genotyping data, but PLINK has low power in detecting shorter haplotypes IBD and GERMLINE has a high false positive rate when trying to detect smaller shared regions [16].

BEAGLE-IBD simultaneously models linkage disequilibrium (LD) between markers and IBD sharing using a Hidden Markov Model, and showed significant improvements in power over earlier programs [16]. However, its computational burden prevents it from being applied to large samples. Having to determine the prior IBD probability is also a potential issue in trying to balance detection power and false positive rate, since suitable priors may vary among populations that differ in history and consanguinity. BEAGLE fastIBD can be applied to large samples while achieving power close to that of BEAGLE IBD [17]. The program still only works on sharing at a pair-wise level and does not provide a comparison between cases and controls to detect haplotype sharing that is associated with diseases.

Several more recent algorithms begin to expand inference of haplotype-sharing IBD from pair-wise sharing to that among multiple individuals. DASH and EMI builds upon pair-wise haplotype sharing to infer groups of individuals who are likely to share a single haplotype [18]. A Markov Chain Monte Carlo (MCMC) probabilistic model applied to unphased SNP data to simultaneously infer haplotype sharing IBD among multiple individuals (MCMC_IBDfinder) was also reported [19]. Based on an embedded log-likelihood ratio and using a model that accounts for LD, Parente2 explicitly models haplotype frequencies and enables large-scale IBD detection [20]. IBD-Groupon makes use of pair-wise IBD relationships detected by other software to infer group-wise haplotype sharing, overcoming the drawbacks of some of the available tools [21]. DASH was used to test diverse GWAS data and showed a significant power increase upon single SNP association, but the power of the algorithm in detecting haplotypes shared IBD by a very small number of cases and/or short haplotypes is unclear. In addition, the power of DASH and IBD-Groupon is dependent on the power of detecting pair-wise haplotype sharing. MCMC_IBDfinder has good power in detecting short shared haplotypes, but it does not provide a comprehensive evaluation process using controls, and is not efficient enough to be applied to GWAS data. The detection power and speed of EMI and IBD-Groupon very much rely on the performance of BEAGLE.

Thus, despite significant progress in developing algorithms to infer haplotypes shared IBD among cases, many problems remain and a comprehensive program applicable to case–control data and with adequate power and efficiency is still lacking. Here we introduce a new algorithm, implemented in HaploShare, designed to have high power when detecting extended haplotypes shared by multiple individuals and the efficiency to be applied to large data sets. Most importantly, it extends an evaluation process using controls to derive an empirical null distribution for the population of interest, to help identify sharing in cases that may harbor disease-associated rare variants.

## Results

### Factors affecting detection sensitivity

**Figure 1:**
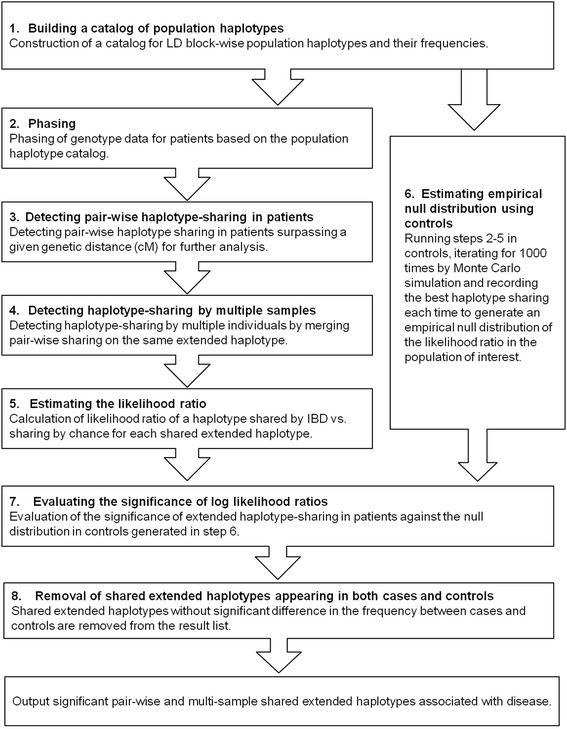
**Flow chart of the steps taken by HaploShare.**

Different scenarios of population-based association studies were simulated by dividing the Hong Kong ethnic Chinese samples randomly into ‘case’ and ‘control’ groups of 1,000 each, and creating recent founder haplotypes of different ages (generations) that were shared by different numbers of ‘cases’ but not by ‘controls’. In all the simulations, a positive detection was defined as the simulated recent founder haplotype reaching a significance of 0.05 (which refers to quasi *P* value, defined in Methods, step 7), based on the empirical null distributions of log-likelihood ratio of sharing by IBD vs. random chance that was derived from haplotype sharing from controls (step 6 in Figure 1).

**Figure 2:**
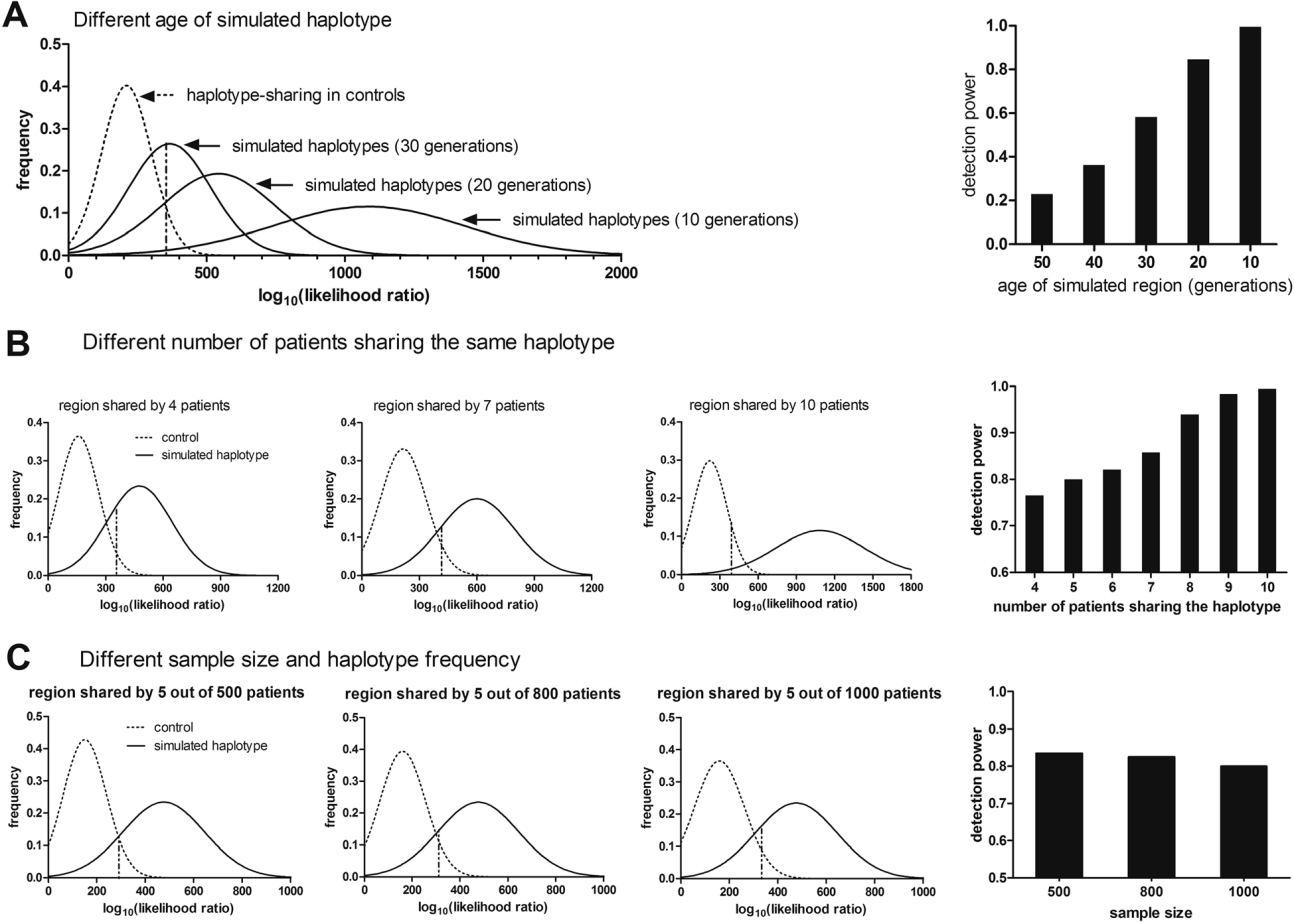
**Detection of simulated founder haplotypes.** The dashed curves are the null distributions of log likelihood ratio derived from controls (step 6, Figure 1) and the solid curves are those of the simulated founder haplotypes. The dashed line perpendicular to x-axis represents the cutoff based on the separation of 95/5% of area under curve of the null distribution. **(A)** Effect of the age of the simulated founder haplotypes on detection sensitivity. The left panel shows the null distribution of the log likelihood ratio from controls (dashed curves) and the simulated founder haplotypes of 10, 20, and 30 generations, respectively (solid curves). The bar chart on the right is the detailed adjusted detection power for founder haplotypes of different ages (generations). The simulations were based on 10 individuals sharing an ancestral haplotype among a total of 1,000 individuals. Data on 1,000 individuals were used as controls. **(B)** Effect of the number of individuals sharing a simulated founder haplotype on detection sensitivity. The left three figures show the separation of log likelihood ratios for the recent ancestral haplotypes from that of the controls when four, seven, and 10 individuals among 1,000 cases share an ancestral haplotype IBD of 10 generations, respectively. They were also evaluated against 1,000 controls. The bar chart on the right shows the detailed detection sensitivity difference by the number of individuals sharing a common ancestral haplotype. **(C)** Effect of the total pool of cases considered on detection sensitivity. The three figures on the left show results when five individuals sharing a common recent ancestral haplotype of 10 generations in age, and are evaluated in a pool of 500, 800, and 1,000 cases, respectively. In each case, 1,000 individuals were used as controls in the evaluation process. The bar chart on the right shows the detection sensitivity in the three different scenarios.

These results indicate that both the age of the founder mutation and the number of cases sharing the same founder haplotype affect detection of the haplotypes. When a threshold of 1 cM was used to select pair-wise haplotype-sharing (step 3 of the algorithm), and 10 of 1,000 individuals carried the founder mutation, HaploShare had an adjusted detection power of almost 1 for mutations 10 generations old. This dropped to 0.23 for 50-generation-old mutations (Figure 2A, right panel). For a 10 generation founder mutation the detection sensitivity was 0.99 if 10 cases shared the haplotype, but 0.76 if only four of the 1,000 ‘cases’ did (Figure 2B). The overall size of the case sample pool considered, however, had little effect on adjusted detection power. For five cases sharing an ancestral haplotype of 10 generations in a pool of 500, 800, or 1,000 total individuals, detection sensitivity remained almost unchanged (Figure 2C), reflecting the robustness of the algorithm against heterogeneity. This is an important characteristic of the algorithm as its purpose is to detect very rare variants associated with the underlying disease.

### Power comparison between HaploShare and other programs

As BEAGLE IBD, PLINK, and GERMLINE only detect pair-wise haplotype sharing without controls, detection sensitivity was compared on haplotypes shared pair-wise, ignoring control data for this purpose. Five out of 100 individuals were assigned founder haplotypes of 10 to 50 generations old for the simulation. These five individuals carry five founder haplotypes, or 500 haplotypes in total over 100 iterations of the simulation. Haplotypes detected as shared by multiple individuals by HaploShare were measured by the number of individuals detected as sharing a founder haplotype (if four shared an extended haplotype, then four are considered found and one is considered missed for the simulation). In addition, steps 1 to 3 only of the HaploShare algorithm (Figure 1) were also examined without performing the multiple sharing evaluation for comparison on detection of pair-wise sharing. Only 100 individuals were used due to the computational demand of BEAGLE IBD. For comparison with DASH, as suggested by the program, we provided pair-wise sharing results from GERMLINE as input in calculation of detection of shared haplotypes. For comparison with IBD-Groupon, the pair-wise sharing results from BEAGLE fastIBD were provided as input. For all the programs, detection was defined as the simulated founder haplotype surpassing a preset threshold for selecting a region, without reference to controls. The parameters used for these programs were described in the Additional file. All the adjusted power was shown in Table 1. We also calculated the 95% confidence intervals of the adjusted power by bootstrap resampling in R (Additional file 1: Table S2).

**Table 1:** **Comparison of adjusted power on detecting pair-wise haplotype-sharing IBD between HaploShare, BEAGLE, GERMLINE, PLINK, DASH, and IBD-Groupon**

**Age of simulated regions (generations)**	**Length (cM)**		**HaploShare (multiple)** ^**b**^		**HaploShare (pair-wise)** ^**c**^		**BEAGLE IBD**	**BEAGLE fastIBD**	**PLINK**	**GERMLINE**	**DASH plus GERMLINE**	**BEAGLE plus IBD-Groupon**
		**Threshold** ^**a**^	**1 cM**	**0.5 cM**	**1 cM**	**0.5 cM**	**N/A**	**N/A**	**0.5 Mb**	**0.5 cM**	**0.5 cM**	**N/A**
50	1.14		45.1	47.8	30.2	38.7	36.6	30.8	0.0	41.9	36.4	29.2
40	1.28		51.9	54.6	35.9	46.9	41.4	40.8	0.0	44.7	40.3	39.3
30	1.53		94.8	95.1	66.6	81.4	49.2	48.8	19.1	49.2	47.7	49.2
20	2.35		97.7	97.8	80.0	90.1	73.2	73.9	39.2	61.1	62.3	73.7
10	4.18		100.0	100.0	94.6	96.9	97.1	96.2	65.6	70.1	72.6	96.5

All results shown here are detections of pair-wise sharing reaching threshold based on respective thresholds in respective programs, no comparison to controls was involved.

^a^For HaploShare, GERMLINE, DASH, and PLINK, it is the minimum genetic distance (or physical distance) of the haplotypes shared pair-wise to be selected for further analysis; for BEAGLE, it is the prior IBD probability; for BEAGLE fastIBD, it is the fastIBD score, and for IBD-Groupon, it is also the fastIBD score.

^b^Haplotypes shared pair-wise surpassing a certain genetic distance (0.5 or 1 cM) are further analyzed for sharing by multiple individuals, which by itself increases power of detecting more individuals sharing an extended haplotype (Step 4, Figure 1).

^c^Analysis stops at only detecting haplotypes shared pair-wise without merging pair-wise sharing into sharing by multiple individuals (stops at Step 3, Figure 1).

It is worth noting that the method for calculation of detection power described above was not directly counting iterations, but counting the fraction of individuals being detected in each iteration. Meanwhile, the false positive rate for each program was not strictly controlled in the power comparison. Therefore, we used the term ‘adjusted power’ instead of ‘power’ in the description of the results. We also used an alternative evaluation method, in which the power was calculated only based on the iterations when all the individuals who carry the simulated founder haplotypes were being detected, while missing any individual with the simulated founder haplotype was defined as a negative detection. The result of this alternative method was presented in Additional file 1: Table S1. It can be seen that very low detection power was observed for short haplotypes for all the programs evaluated using this method.

When detecting pair-wise sharing only, HaploShare outperformed other programs for haplotypes of 20 to 30 generations old and this advantage was even greater if multiple sharing was considered (Table 1 and Additional file 1: Table S2). The length cutoff in selecting haplotypes shared pair-wise affected the adjusted detection power of HaploShare. At a cutoff of 0.5 cM instead of 1.0 cM, adjusted detection power increased, especially for founder haplotypes of older age (Table 1 and Additional file 1: Table S3). HaploShare was more powerful than other programs even when only pair-wise evaluation was performed. This indicates that the improved power of HaploShare was not solely based on the multiple sharing analysis scheme.

PLINK and DASH are programs that make use of case control data. Identification of shared haplotypes that reach statistical significance based on the controls by PLINK, DASH, and HaploShare was examined. Two, five, or 10 out of 1,000 individuals were assigned founder haplotypes of 10 to 50 generations of age. HaploShare showed higher adjusted power in detecting shared extended haplotypes reaching significance (Table 2 and Additional file 1: Table S4, S5) as well as simulated haplotypes reaching detection threshold (Table 1). However, these data also showed that a number of the simulated founder haplotypes were detected but did not reach statistical significance.

**Table 2:** **Adjusted power and rank of the simulated founder haplotypes among all the regions found by HaploShare, BEAGLE fastIBD, and DASH**

**Software**		**HaploShare**						**BEAGLE fastIBD**		**DASH + GERMLINE**		
Threshold		Pair-wise IBD >1.0 cM, *P* <0.05			Pair-wise IBD >0.5 cM, *P* <0.05			fastIBD score <10^−10^	<10^−12^ 15 found	Pair-wise IBD >0.5 cM		
Total number of haplotypes found^a^		5 found			32 found			139 found		89 found		
Number of samples sharing the simulated haplotype		2	5	10	2	5	10	2	2	2	5	10
Age of simulated founder haplotype (generations)	10	1 (74)	1 (83)	1 (99)	4 (91)	3 (95)	3 (99)	32 (95)	11 (35)	50 (65)	46(75)	38 (82)
	20	1 (60)	1 (78)	1 (86)	10 (73)	8 (85)	6 (90)	45 (69)	13 (21)	60 (53)	57 (65)	54 (71)
	30	2 (34)	2 (47)	1 (60)	20 (50)	19 (64)	15 (72)	88 (41)	14 (16)	68 (41)	65 (49)	64 (52)
	40	3 (19)	3 (26)	2 (39)	24 (39)	23 (47)	21 (53)	107 (34)	15 (12)	75 (35)	75 (39)	75 (45)
	50	5 (09)	4 (13)	3 (23)	27 (32)	27 (39)	27 (41)	125 (28)	15 (11)	76 (30)	80 (33)	80 (37)

Shown are the rank and (detection power %) of the simulated haplotypes.

^a^Total extended haplotypes found are an average of all the extended haplotypes shared reaching significance from 100 simulations, which usually (depending on power) includes one simulated founder haplotype shared by two, five, or 10 samples in each simulation. The total sample size in each simulation is 1,000 and haplotypes shared by 20 samples (2%) or fewer were analyzed. When 1 cM was used as the threshold for selecting haplotypes shared pair-wise, there were five haplotypes reaching significance on average, including the simulated founder haplotype. When 0.5 cM was used as the threshold, there were 32 haplotypes reaching significance. Any of the haplotypes found repeatedly in different simulations were only counted once.

To address a potential issue that might arise from the similarity in the simulation of the shared founder haplotypes and the algorithms used in their detection by HaploShare, an alternative set of founder haplotypes was also generated (described in Methods). With shared haplotypes by siblings phased by BEAGLE serving as the shared recent founder haplotypes, HaploShare achieved comparable adjusted power to when using founder haplotypes generated by its own algorithm (Additional file 1: Table S6), indicating that generating and detecting founder haplotypes both by HaploShare did not cause inflation in adjusted power. This alternative set of founder haplotypes were also used to test other tools and the results were consistent for founder haplotypes generated by different methods (Additional file 1: Table S7). Similarly, we have done testing and evaluation of HaploShare and other software using WTCCC data, a widely used data source from subjects of European ancestry. Comparable results were achieved (Additional file 1: Table S8), indicating that the performance of the various programs is not dependent on the data and is not population-specific.

### Evaluation of false positive detections

Extended haplotypes that were shared by a number of case individuals but without intrinsic relationship with the underlying disease may still reach significance and were considered false positive detections for the sake of testing the different tools, even though some of them might be true IBD derived from a recent common founder. Log likelihood ratios of sharing by IBD vs. chance for extended haplotypes detected from control samples (see Methods) follow a shifted Gamma distribution (solid curve, Additional file 1: Figure S2). A distribution of the largest likelihood ratios (the extreme values) derived from iterations by repeated applications of this process was obtained and it follows a Gumbel distribution. The right tail to the *P* = 0.05 point of this distribution (dashed curve, Additional file 1: Figure S2) was used to obtain a cutoff for the log likelihood ratio of an extended haplotype. At a selection threshold for pair-wise sharing of 1 cM, on average 7.3 × 10^5^ extended haplotypes shared by two to 20 individuals among a pool of 1,000 people were found and evaluated, and on average, nine surpassed the quasi *P* value cutoff in 100 repeats. Comparing these shared haplotypes in cases with those by controls showed that on average 4.4 of them were likely to be shared by both cases and controls without significant frequency difference between the two groups, and they were removed from further consideration, leaving 4.6 false positives on average. This gave an empirical false positive rate of 6.28 × 10^−6^ (Additional file 1: Table S9). The false discovery rate (FDR) depends on the ratio of shared extended haplotypes associated with the underlying disease and those that are not, which is an unknown parameter. Assuming 10,000 or 1,000 non-disease associated shared haplotypes would be evaluated for each disease associated haplotype with significant quasi *P* value, then FDR was calculated as 0.059 or 0.006, respectively. Reducing the threshold for selection of pair-wise sharing from 1 cM to 0.5 cM increased adjusted power but caused a slight increase in the false positive rate and FDR (Additional file 1: Table S9).

We also compared the adjusted power of HaploShare with BEAGLE fastIBD + IBD-Groupon under the same level of false positive rate. Parameters from HaploShare and BEAGLE were adjusted so that the same number of non-simulated findings were detected, which made the false positive rate identical between the two methods. Summarized results showed that when controlling the false positive rate, HaploShare still demonstrated superb power against BEAGLE fastIBD + IBD-Groupon (Additional file 1: Table S10).

An important concern is how many such shared extended haplotypes will be found and, what would be the rank of the recent founder haplotypes associated with diseases among all such regions found. Under the conditions above, the ranks of simulated founder haplotypes shared by two, five, or 10 samples (0.02%, 0.05%, and 1% of the cases) among all haplotypes reaching the threshold were in the range of 1 to 5 on average from the 100 simulations (Table 2 and Additional file 1: Table S11), depending on the age of the haplotype and the number of the individuals who shared it. This was significantly better than BEAGLE fastIBD and DASH tested on the same dataset, which either detected too many regions and ranked poorly the simulated founder haplotypes (BEAGLE fastIBD using fastIBD score of 1 × 10^−10^ and DASH using pair-wise results from GERMLINE and pair-wise IBD >0.5 cM), or had unacceptable power if 1 × 10^−12^ was used as the fastIBD score (Table 2).

For HaploShare, 1 cM as selection threshold for extended haplotypes shared pair-wise gave a good balance of adjusted power and false positive detection. At a threshold of 0.5 cM, many more extended haplotypes reached significance (32 vs. 5), and the simulated founder haplotypes had much lower ranking among all the significant haplotypes detected. A significant gain in adjusted power was observed using 0.5 cM as the threshold (Table 2) if small numbers of individuals (for example, two or five of 1,000) shared a founder haplotype, but little if more did (for example, 10).

### Computing efficiency

To investigate computing efficiency, 100 samples were selected as cases for both HaploShare and other programs. An additional 500 samples were provided for HaploShare as controls. GERMLINE, PLINK, and BEAGLE fastIBD took 2, 3, and 4 min, respectively, and DASH took 3 min processing results from GERMLINE, to process the 100 samples while BEAGLE IBD took nearly 5 days. HaploShare took 29 h to finish dealing with 100 cases and repeatedly (1,000 times) drawing 100 samples from controls to generate the empirical null distribution of extended haplotype sharing. The major computationally expensive step for HaploShare was to combine pair-wise shared haplotypes into sharing by multiple individuals, a process not adopted by other programs. Detection of pair-wise sharing by HaploShare took 5 min for 100 cases, comparable to the other faster programs (Table 3).

**Table 3:** **Comparison of computing time between HaploShare, BEAGLE, GERMLINE PLINK, and DASH**

**Input data**	**HaploShare**	**BEAGLE (IBD)**	**BEAGLE (fastIBD)**	**GERMLINE**	**DASH plus GERMLINE**	**PLINK**
	**(100 cases, 500 controls)**	**(100 cases)**	**(100 cases)**	**(100 cases)**	**(100 cases)**	**(100 cases)**
Phased genotype	21 h	115 h	4 min	2 min	5 min	N/a
Unphased genotype	29 h	116 h	38 min	N/a	N/a	3 min

There is a significant increase in computing time if the threshold for selecting pair-wise shared haplotypes is reduced. A cutoff of 0.5 cM takes three times as long to compute as a cutoff of 1 cM. On a larger dataset with 500 cases and 1,000 controls, nearly 88 h were required to finish the analysis. The time complexity is approximately quadratic in sample size (Additional file 1: Table S12). The test was run on a computer with Intel® Core™2 Quad Processor Q9650 and 8 GB of RAM.

### Detection of a known recent founder haplotype (real data)

A R114H mutation in the Ret proto-oncogene (*RET*) is known to be associated with Hirschsprung’s disease and shared by a small number of Chinese cases [22]. This mutation was not found in any of the Chinese controls, or in Caucasian cases or controls. An ancestral 250.2 kb haplotype on which the mutation resided was found to be shared by all the cases who carried the R114H *RET* mutation but none of the non-carriers, indicating that this mutation was probably derived from a recent founder [22].

Data on 173 Hirschsprung’s disease cases and 436 controls, all of whom Chinese ethnicity and genotyped by an Affymetrix 500K chip, were analyzed. Based on the original study, 14 of the 173 cases carry the R114H *RET* mutation. Evaluation by HaploShare showed that the extended haplotype on which the R114H *RET* mutation resided had a significant quasi *P* value of 0.004. Several other extended shared haplotypes reached empirical significance (31 regions with quasi *P* <0.05 and nine regions with quasi *P* <0.01), which could be additional recent founder haplotypes still to be confirmed, or false positive results caused by population stratification or random chance. The haplotype that the R114H *RET* mutation resided on ranked fourth among all the significant haplotypes.

**Figure 3:**
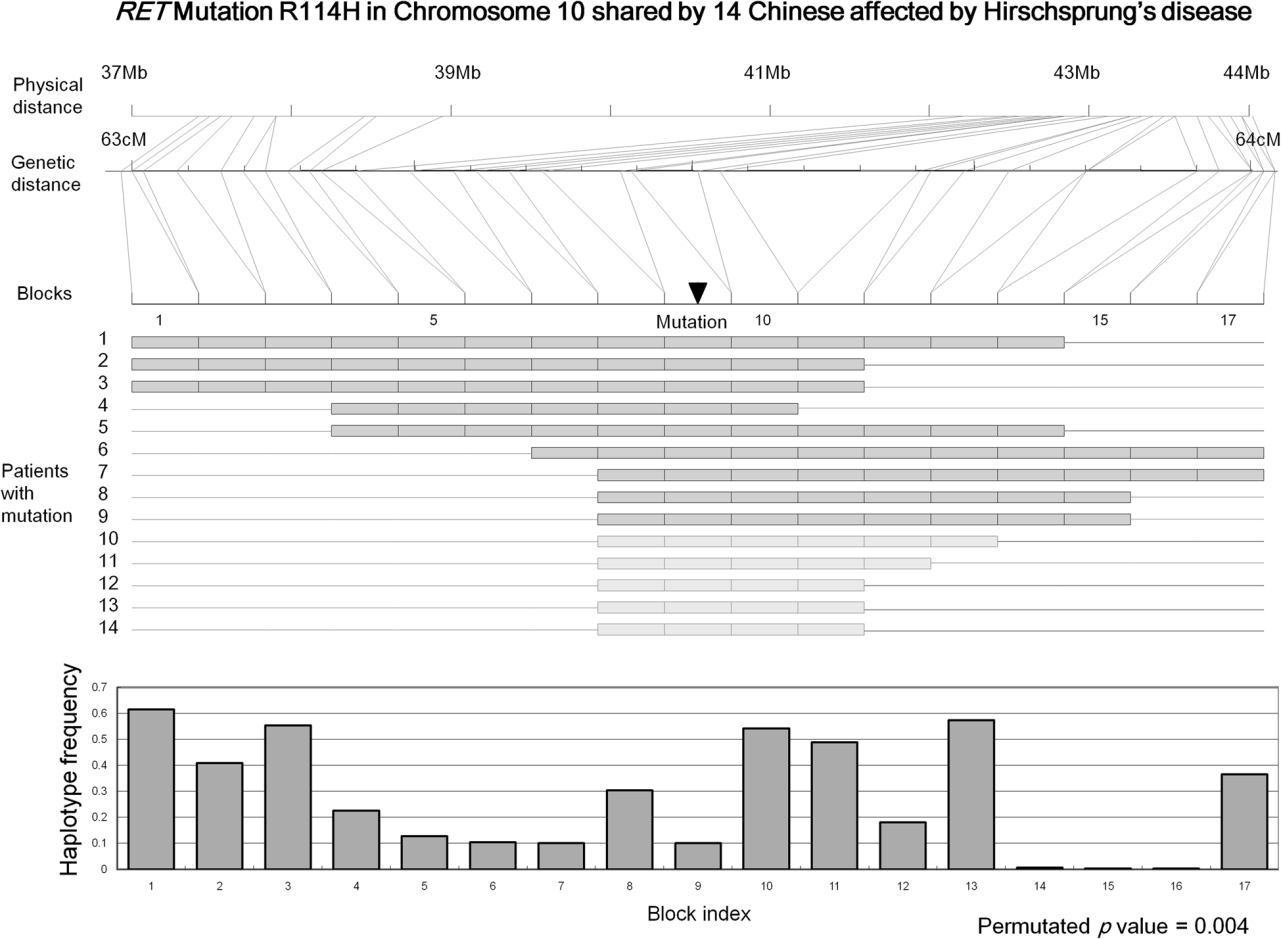
**Illustration of an extended haplotype shared by multiple individuals.** In this example, four individuals share an extended haplotype in a region composed of 23 SNPs with six LD haplotype blocks. Two blocks form the core haplotype that is shared by all four individuals. The shared haplotype in each LD block (middle panel) and their frequencies (lower panel) are displayed below each block. For block 5, three samples share both haplotypes. In this case, the haplotype with a higher frequency is used during the evaluation process.

For the *RET* locus, a shared haplotype of about 7 Mb, much bigger than the reported haplotype in the original study, was detected although the region has a genetic distance of about 1 cM only (Figure 3). Nine of the 14 cases carrying the mutation were identified directly using a threshold of 0.5 cM for selecting pair-wise shared haplotypes. Five additional cases were identified through further loosening the inclusion criterion to 0.25 cM, a built-in process of HaploShare for regions with confirmed sharing of extended haplotypes. The core haplotype region shared by all 14 cases spanned about 220 kb, consistent with the original report [22].

## Discussion

Next-generation sequencing technology greatly accelerated the pace of novel mutation detections. However, firmly establishing a link between detected variants and an underlying disease is still difficult, largely due to the low allele frequencies of the variants and the limited number of individuals that can be sequenced. Detection of extended haplotypes that are shared potentially IBD among cases can help evaluate the rare variants found. Conversely, the individuals and regions suggested by such analyses can serve as candidates for next-generation sequencing, focusing resources on a limited number of individuals and/or regions that share a potential founder haplotype.

The HaploShare algorithm presented here facilitates the detection of haplotypes potentially shared IBD among multiple individuals, rather than only pairs, from a case group based on equivalent analyses in a control group. Haplotypes shared among small numbers of individuals (<2% as default cutoff) are evaluated by log likelihood ratios of shared as IBD vs. shared by chance. By comparing these ratios to a distribution of maximal values from those equivalently analyzed in a control group, statistically significant extended haplotype sharing in cases can be identified with good power and reasonable computational speed.

These features of HaploShare lead it to outperform existing programs, showing greater adjusted power and reduced false positives, while maintaining tractable computational performance. By recording all possible haplotype pairs that can explain block-wise genotypes and all possible transition between blocks, HaploShare can overcome phase uncertainty that may have affected the power of algorithms based on Hidden Markov Model, which may account for most of its improvement in sensitivity. HaploShare is more computationally intensive than PLINK, GERMLINE, DASH, and BEAGLE fastIBD, but delivers multiple shared haplotypes, rather than only pair-wise sharing. Its computational performance is much better than BEAGLE-IBD. Most importantly, direct comparison to the best sharing in controls help identify sharing that is likely associated with an underlying disease.

HaploShare requires the control sample size to be larger than that of the cases. If this is not practical, HaploShare can generate virtual samples according to LD between blocks, SNP allele frequencies and block-wise haplotype frequencies of existing samples or those from HapMap or other studies. Many of the measures used to generate empirical null distributions of shared haplotypes reflected the compromises made due to lack of huge number of controls in practice. An alternative approach is to treat cases that do not carry the haplotype under evaluation as controls. This not only increases the availability of control samples, but also provides an additional measure that can reduce the false positive rate if there is a population substructure mismatch between cases and controls. Although homogeneous populations are not essential for HaploShare, good matching of cases and controls is desirable. This was highlighted in the WTCCC Bipolar disorder dataset where greater case-case IBD than control-control IBD was found, probably due to different geographical origins between the cases and controls [16].

Even in an outbred population, various cryptic relationships may exist. This may complicate the evaluation of extended haplotype sharing and presents a major source of false positive detections. HaploShare adopted a non-region-specific approach to evaluate sharing of extended haplotypes genome-wide, based on the likelihood ratios of sharing IBD vs. by chance to reduce false positive detections. To supplement this approach, an additional step of region-specific comparison between cases and controls was used to compare the detected regions between cases and controls, to distinguish those specific to a case group from the ones existing at the similar level in the general population. This approach significantly reduced false positive detection with little effect on power based on simulation results (Additional file 1: Table S5).

Increases in SNP coverage over that of the Illumina Beadchip 610-Quad used in the examples here, such as by making use of rare variants identified by either the 1000 Genome Project or other whole genome or exome sequencing efforts (like the exon-chip) will likely increase the power of detection. Thus, identification of shared ancestral haplotypes of much older age or shared by fewer individuals than demonstrated here should be possible. With modest modification, HaploShare can be made applicable to detection of potential haplotype-sharing IBD on the same locus but on different alleles by different pairs, to identify disease loci where allelic heterogeneity may exist. If parallel computing is implemented, HaploShare is capable of working on thousands of samples within days. HaploShare extends our ability to evaluate extended haplotypes shared among cases that are associated with diseases with a computationally practical procedure.

## Conclusion

We have developed a novel algorithm and software implementing it, HaploShare, for detecting shared ancestral haplotypes by multiple individuals, evaluated by control data from the same population. HaploShare tackles the issue of phase uncertainty by utilizing population haplotype information and considering any combinations of haplotypes that can explain genotypes in a region. It is efficient enough to apply to large scale GWAS data. Comparison of HaploShare and other methods on both simulated and real data demonstrated its improved power and reduced false positive rate.

## Materials and methods

A flow chart of the seven-step processes that HaploShare uses in detecting and evaluating haplotype-sharing IBD is shown in Figure 1.

### Step 1. Construction of a catalog of population haplotypes

The entire genome of each control subject is divided into regions of LD blocks and recombination hotspots based on LD between SNPs in healthy controls, using a modification of an earlier method [23]. A pair of SNPs is considered to have strong or weak LD if the one-sided upper 95% confidence bound of D’ is >0.98 or <0.90. Pairs between these values are not considered. If (‘strong LD’ pairs) / (‘strong LD’ pairs + ‘weak LD’ pairs) >0.95 in a region, it is considered an LD block. Otherwise it is treated as a recombination hotspot and these SNPs will be considered individually. Evaluation of LD blocks and hotspots starts from the most 5′ SNP in a chromosome. Once the first pair of adjacent SNPs with strong LD is identified, downstream SNPs are added to the block by evaluating the LD of each addition with all SNPs in the region, until the definition of an LD block is no longer met or two adjacent SNPs are separated by >50 kb. PHASE software [24,25] is then used to generate a catalog of haplotypes for each predefined LD block together with their population frequencies.

If no control data are available, LD blocks and recombination hotspots are defined from genetic distances between SNP markers obtained from HapMap. A genetic distance smaller than 0.001 centiMorgans (cM) between two adjacent SNPs within 10 kb extends an LD block. The two methods usually generate similar results of LD blocks and recombination hotspots (Additional file 1: Figure S1). Phased data from the corresponding population in HapMap will be used to generate a catalog of haplotypes in this case.

### Step 2. Phasing of genotypes in cases based on a population haplotype catalog

Genotypes in cases in each LD block are phased into haplotypes based on the population haplotype catalog. Several scenarios may apply. (A) Only one pair of haplotypes in the catalog can explain the genotypes in a block. (B) If more than one pair of haplotypes is consistent with the genotypes in a block, all the possible pairs of haplotypes will be recorded and used in later steps in identifying haplotype sharing. (C) The genotype can only be explained by a known haplotype together with a haplotype that does not exist in the catalog. A low population frequency, which is estimated as

$$ {h}_u<1-\sqrt[2m]{0.95} $$

is assumed for the undetected haplotype. Here *m* is the sample size of the controls used to generate the haplotype catalog and a 95% probability of a haplotype being absent in the controls is assumed. (D) The genotypes are not consistent with any of the known haplotypes in the catalog. In this case, genotyping errors are assumed and the LD block will not be used for initial detection of haplotype sharing. However, it will not prevent extension of a shared haplotype that contains it.

### Step 3. Detecting pair-wise haplotype-sharing in cases

After obtaining phasing results for all the cases, pairs of individuals are scanned and compared for potential haplotype sharing across each chromosome. Only haplotypes shared in an LD block (not SNPs in recombination hotspots) are used to initiate matching. The shared region is extended in both directions while supported by haplotypes in LD blocks or SNPs in hotspots, allowing for potential genotyping errors. Genotyping errors are tolerated if 1% or fewer SNPs in an extended haplotype do not comply with haplotype-sharing. No incompatibility is allowed in the first and the last 20 SNPs of an extended shared haplotype to avoid false extensions. Extended haplotypes shared pair-wise longer than a preset genetic distance (such as 0.5 or 1 cM as used in the simulations shown in this study) are selected and evaluated for sharing among multiple individuals.

### Step 4. Detecting haplotype-sharing by multiple samples

**Figure 4:**
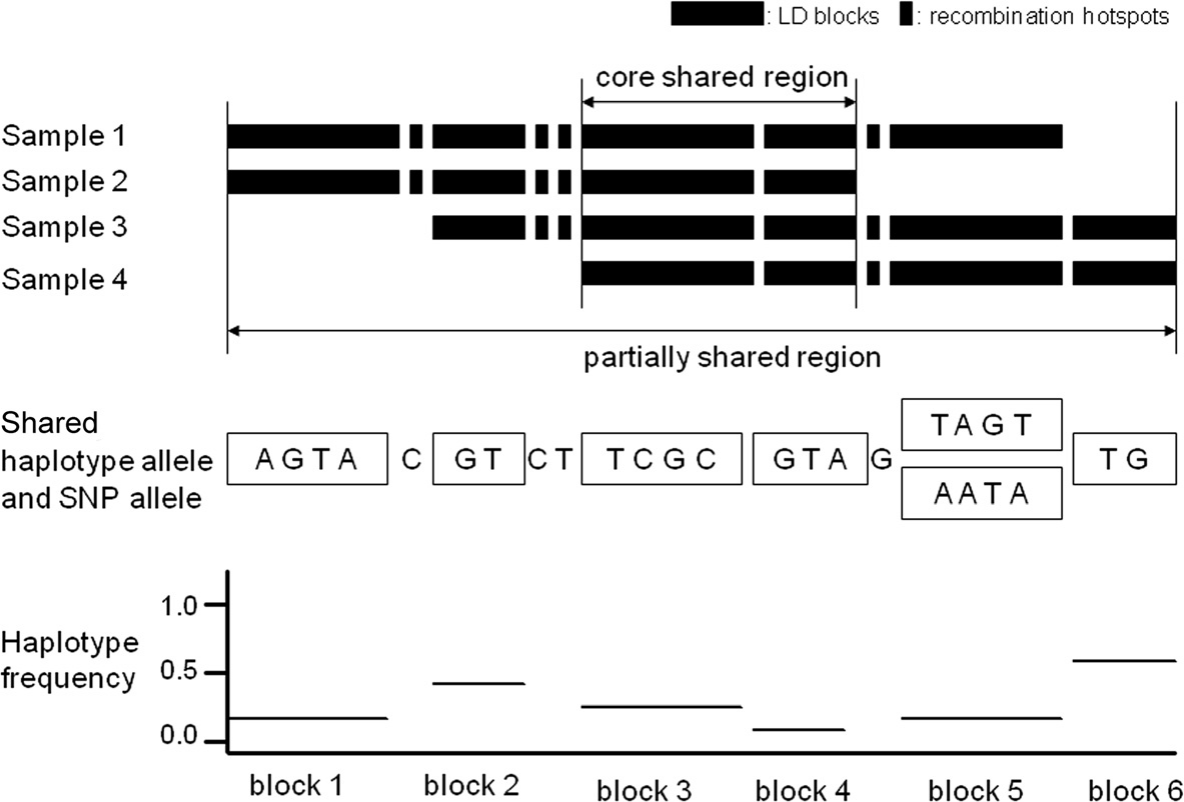
Detection of the haplotypes carrying the RET mutation. The RET mutation region spans 7 Mb in physical distance but only 1 cM in genetic distance, which contains 17 LD blocks. All 14 cases share the core haplotype composed of blocks 8 to 11; and the rest of the region is shared by at least two cases at any given point. The frequency of the shared haplotypes in different blocks varies from less than 1% to 60% among the Hong Kong Chinese population.

Haplotypes shared by pairs are compared with each other to identify those that share at least part of an extended haplotype. They are merged when different pairs overlap at least partially over the shared region on the same allele. An extended haplotype shared by multiple individuals is defined as a haplotype that is shared by at least two individuals at any given point and contains a core haplotype that has to cover at least one LD block and be shared by all the individuals. An example of this is illustrated in Figure 4. The method may miss individuals who share a portion of the extended haplotype but did not meet the selection threshold for pair-wise sharing with any other samples in this group. To include these in further analysis, the remaining samples are scanned for those that share the entire core haplotype and have a total length of shared haplotype that is at least half the threshold initially used (see step 3 above).

### Step 5. Estimating the likelihood ratio of a haplotype shared by IBD vs. sharing by chance

Most shared haplotypes from the general population are short and common, although physical length can be a poor indicator [26]. True recent founder haplotypes, however, are expected to be longer and may contain multiple block-wise haplotypes of low frequency in the population. Here we introduce an estimate of the likelihoods of sharing due to IBD or by chance and use the logarithm of their ratio to evaluate haplotype sharing events.

If sharing is due to random chance, then inheriting haplotypes from different blocks should be independent events, disregarding weak LD between blocks. Thus, its likelihood can be estimated as the product of the likelihoods of inheriting haplotypes in different LD blocks. The probability of sharing haplotype *i* in LD block *j* by random chance by *k*_*j*_ individuals is designated as *P*_*j*_, which, based on the population frequency of the shared haplotype and the number of individuals who share the haplotype, can be estimated as:

$$ {P}_j={h_{ji}}^{k_j}\cdot \frac{{\left(\begin{array}{c}\hfill 2\hfill \\ {}\hfill 1\hfill \end{array}\right)}^{k_j}}{\left(\begin{array}{c}\hfill 2k\hfill \\ {}\hfill k\hfill \end{array}\right)} $$

The population frequency of haplotype *i* in block *j* is *h*_*ji*_. Here *k* is the number of individuals who share the extended haplotype, and *k*_*j*_ is the number of individuals in *k* that share haplotype *i* in block *j* and *k*_*j*_ ≦ *k*. $$ \left(\begin{array}{c}\hfill 2k\hfill \\ {}\hfill k\hfill \end{array}\right) $$ stands for the number of possible combinations of *k* chromosomes from a total of 2 *k* chromosomes, and $$ {\left(\begin{array}{c}\hfill 2\hfill \\ {}\hfill 1\hfill \end{array}\right)}^{k_j} $$ indicates that one of the two haplotypes is shared by each of the *k*_*j*_ individuals. The likelihood of sharing the extended haplotype by random chance is:

$$ L\left({H}_0\right)=\left(\begin{array}{c}\hfill s\hfill \\ {}\hfill k\hfill \end{array}\right)\cdot {\displaystyle \prod_{j=1}^m{P}_j} $$

where *s* is the total number of individuals considered (for example, the sample size of cases), and *m* is the total number of LD blocks within this extended shared haplotype. Thus *L(H*_o_*)* represents the probability of *k* individuals sharing an extended haplotype by random chance, and the extended haplotype has *m* LD blocks with respective haplotype frequencies of *h*_*ji*_. Only block haplotypes are considered for the likelihood estimation.

If the region is shared due to inheritance from a recent common ancestor, the likelihood can be estimated as a function of the estimated age, n, of the shared haplotype (generations) based on the genetic length of the region and k, the number of individuals who share the extended haplotype:

$$ L\left({H}_1\right)={0.5}^{n\cdot k} $$

So *n* × *k* represents the total number of meioses in shaping the shared extended haplotype and 0.5 is the chance that the haplotype is inherited in each meiosis. Since different individuals may share different lengths of the extended haplotype, *n* is estimated by the average genetic distance *d*_*g*_ (cM) of the shared haplotype for each of the *k* individuals:

$$ E\left({d}_g\right)=\frac{100}{n} $$

An inherited haplotype of 2 cM on average indicates that approximately 50 meioses may have occurred in the surrounding region, suggesting sharing of a common ancestor about 50 generations ago. The logarithm of the likelihood ratio of sharing by IBD and sharing by chance is then estimated as:

$$ { \log}_{10}\frac{L\left({H}_1\right)}{L\left({H}_0\right)} $$

### Step 6. Estimating empirical null distribution using controls

The likelihood ratio may be inflated due to weak LD between haplotype blocks. Differences in coverage over the genome, especially on rare genetic variants may introduce inaccuracies. Thus a genome-wide correction is necessary to make the likelihood ratio comparable across different regions. A Monte Carlo simulation process is used to survey the distribution of shared haplotypes in healthy controls.

If a region is shared by *k* individuals among a total of *s* cases, then *s* samples are selected randomly from the total pool of controls. For these individuals, haplotype-sharing is detected and the likelihood ratios of sharing by IBD and sharing by chance are calculated as described above. In each simulation, the largest likelihood ratio from all the extended haplotypes in the entire genome shared by *k* or fewer individuals is recorded. The reason of using the largest likelihood ratio from each iteration to calculate the null distribution in a population rather than using those from all the extended haplotypes evaluated is a reflection of the difficulty of estimating the null distribution of the likelihood ratio while taking the multiple testing issue into consideration. Since it is very difficult to model the effect on likelihood ratios by the age of the extended haplotype and the number of individuals sharing it, this constraint ensures that the extended haplotypes in cases that are more recent in history are detected. This process is repeated at least 1,000 times by selecting different set of samples from controls by random. If the available samples in the controls are smaller than 2 *s*, virtual samples are generated according to the SNP allele and block haplotype frequencies of the existing samples, as detailed below. Each simulation round uses the entire control sample except for the extended haplotypes already selected and recorded, to avoid repeated selection of the same haplotypes among simulations. This is an approach addressing the issue that the control samples are often not large enough to allow repeated simulation without selecting the same subset of individuals between different rounds.

This generates a collection of extreme values of likelihood ratios on haplotype-sharing from controls, which follow a Gumbel distribution, with the goodness of fit always reaching 0.99 or higher under Anderson-Darling test performed in Matlab (*P* >0.01, accept H_0,_ which indicates that the data fit the distribution). Therefore, this distribution is treated as the empirical genome-wide null distribution of the likelihood ratios for haplotype-sharing in the population of interest (shown as dashed curves in Figure 2 and Additional file 1: Figure S2), from which the type I error can be estimated and thresholds determined based on the area under the curve on the right tail.

If control samples are not available or are smaller than twice of that of the cases, HaploShare generates a virtual control data set based on the haplotypes and their population frequencies based on HapMap data or the provided controls. Briefly, for genotypes in blocks, two haplotypes are randomly selected with probability proportional to their population frequency. For genotypes in recombination hotspots, SNP alleles are chosen by a Markov model with the chance of being selected proportional to its weak LD to its 5′ SNP, as described previously [26]. This procedure ensures that the block-wise haplotype frequencies and LD for recombination hotspot SNPs remain similar to those in the original HapMap samples or the limited controls provided.

### Step 7. Evaluating the significance of log likelihood ratios

*P* values are defined as the probability of exceeding the observed statistic under the null hypothesis. As mentioned above, the null hypothesis generated in this method (step 6) was not based on all the extended haplotypes evaluated, but the best ones from the iterations. Therefore, to avoid confusion, we used the term ‘quasi *P* value’ instead of ‘*P* value’ in this method. The ‘quasi *P* value’ is not directly reflecting the false positive rate of the method, but only evaluating the significance of each haplotype sharing. The distribution generated in step 6 was applied to find the ‘quasi *P* values’ of the haplotype sharings identified among the cases. Additional file 1: Table S10 demonstrated the way to calculate the false positive rates from certain quasi *P* values.

### Step 8. Removing the extended haplotypes shared by both cases and controls without significant frequency differences

In order to further reduce false positive findings, significantly shared haplotypes in cases are then compared to those shared in controls to find the ones overlapping in both location and allele, and without significant differences in the number of individuals who share the haplotype. For each shared haplotypes found to be significant in cases, HaploShare examines the samples in all the controls that carry the same haplotype by the following criteria: (1) there is a complete overlap on the core haplotype between sharing in cases and controls; (2) the samples in controls also share over 50% of the entire extended haplotype shared in cases; and (3) there is no significant difference in the frequency of the shared haplotype between cases and controls based on Chi square test (*P* >0.05). The haplotypes shared in cases as well as in controls so defined are considered to be without evidence of association with the underlying disease and are removed from the findings.

### Generation of datasets to evaluate HaploShare and other programs

A real dataset of Hong Kong Chinese samples genotyped using Illumina 610-Quad Beadchips was used to evaluate this methodology (>2,800 individuals; see Additional file for details of the data). The data was first used to evaluate the phasing method introduced in HaploShare. Genotypes from one set of 1,000 individuals were used to generate the block-wise population haplotype catalog and another set of 1,000 individuals was randomly chosen to test the phasing process. About 91% of the markers were found to be located in LD blocks and the rest in recombination hotspots. Ninety-seven percent of the block-wise genotypes could be explained by a unique pair of haplotypes; 2.7% were explained by more than one pair of haplotypes and 0.2% of the block-wise genotypes could only be explained by one known haplotype and an unknown haplotype. Very rarely were block-wise genotypes (<0.01%) not compatible with any known haplotype in this dataset, although this will depend on the size of the control sample used and the error rate of the genotyping platform.

To generate recent founder haplotypes, the meiosis process was simulated as described previously [27]. In each simulation, one sample was selected as an ‘ancestor’ and several samples as the last generation ‘descendants’. The genotypes of the selected samples within LD blocks were phased according to the block-wise haplotype catalog introduced above (or by using genotypes from family members phased by BEAGLE, see below). LD between two adjacent SNPs in recombination hotspots or between LD blocks was modeled as described above. Therefore, LD both within and between blocks was taken into account in the phasing process.

After phasing, a single ‘ancestor’ SNP was randomly chosen as the ‘founder mutation’. In the simulated meiosis process, the extended haplotype carrying the mutation was shortened through each simulated recombination event [27]. Once the range of the haplotype carrying the ‘founder mutation’ was determined, one of the alleles in its location in each of the last generation ‘descendants’ was replaced with the mutation-carrying founder haplotype to generate genotypes carrying this ancestral haplotype. The ‘affected descendants’ created this way were mixed with other randomly chosen individuals in the ‘case’ group for testing HaploShare and other programs. Additional file 1: Figure S3 shows the correlation of the genetic distance and age (generations) of the founder haplotypes simulated in this study.

An alternative method of generating founder haplotypes that is less similar to the algorithm used by HaploShare was also used in this study. It used data from 192 sib-pairs genotyped using Illumina 610-Quad Beadchip. Regions potentially shared IBD between siblings were defined as any region of more than 10 cM in genetic distance and contains fewer than 0.1% of markers with complete genotype mismatch (discordant homozygous genotypes). For each sib pair, 960 such potential IBD regions of various lengths (1, 2, 3, 4, and 5 cM in genetic distance) were randomly selected. Phasing of the IBD regions was performed using BEAGLE [16]. The haplotypes that were shared IBD between siblings were then used as ‘founder haplotypes’ in the simulation process described above and the sensitivity of HaploShare in detecting the founder haplotypes generated by the two different methods was compared.

The false positive rate and the rank of the simulated founder haplotypes among all the significant extended haplotypes shared in the entire genome were evaluated. One thousand individuals were randomly selected from the real dataset of Hong Kong Chinese samples as testing data in each simulation. In 100 replications each, two, five, or 10 of the 1000 individuals were inserted with a simulated recent founder haplotype of 10to 50 generations in age. All shared extended haplotypes (simulated and un-simulated) identified by HaploShare at a significance level of quasi *P* <0.05 were counted and ranked by their quasi *P* values. The average number of shared haplotypes identified and the rank of the simulated founder haplotypes were used to evaluate false positive detection. Due to its design constraints, BEAGLE fastIBD can only use simulated pair-wise sharing of haplotypes derived from these simulations as a test for false positives.

### Data availability

The program and the data used for test the programs are available from the following websites:

1. WTCCC data: http://www.wtccc.org.uk/
2. Hapmap genetic distance map: http://hapmap.ncbi.nlm.nih.gov/downloads/recombination/
3. Hapmap phasing genotype: http://hapmap.ncbi.nlm.nih.gov/downloads/phasing/?N=D
4. HaploShare (windows) and testing data: http://paed.hku.hk/genome/software.html or https://github.com/jonsonfox/HaploShare

## Additional file

Additional file 1: Figure S1. Number of SNPs and the length of the haplotype blocks. **Figure S2.** Estimation of false positive rate. **Figure S3.** Length of pair-wise sharing (cM, overlapped region only) of the simulated IBD regions of different age. Quality control of the testing data. The parameters used for GERMLINE, BEAGLE, DASH, and PLINK. **Table S1.** Alternative comparison of adjusted power on detecting pair-wise haplotype-sharing IBD between HaploShare, BEAGLE, GERMLINE, PLINK, DASH, and IBD-Groupon using an alternative method. **Table S2.** Ninety-five percent confidence intervals of the adjusted power in Table 1. **Table S3.** Comparison of adjusted power on detection of pair-wise haplotype-sharing IBD between HaploShare and others (sort by length). **Table S4.** Ninety-five percent confidence intervals of the adjusted power in Table 2. **Table S5.** Adjusted power of detection on shared extended haplotype significantly associated with diseases. **Table S6.** Adjusted power comparison on founder haplotypes generated by two different methods. **Table S7.** Adjusted power and rank of the founder haplotypes derived from sibpairs among all the regions found by HaploShare, BEAGLE fastIBD, IBD-Groupon, and DASH. **Table S8.** Comparison of adjusted power on detecting haplotype-sharing IBD between HaploShare, BEAGLE, GERMLINE, PLINK, DASH, and IBD-Groupon on simulated data from WTCCC data. **Table S9.** False positive rate and false discovery rate. **Table S10.** Power comparison with same false positive rate. **Table S11.** Ninety-five percent confidence intervals of the rank in Table 2. **Table S12.** A breakdown of time consumed by different steps by HaploShare.
