# Supplementary material for: HaploShare: identification of extended haplotypes shared by cases and evaluation against controls
Source: Genome Biol. 2015 May 9;16(1):92. doi: 10.1186/s13059-015-0662-9 (PMC4432975; doi:10.1186/s13059-015-0662-9)
Supplement: Additional file 1: Figure S1. — Number of SNPs and the length of the haplotype blocks. Figure S2. Estimation of false positive rate. Figure S3. Length of pair-wise sharing (cM, overlapped region only) of the simulated IBD regions of different age. Quality control of the testing data. The parameters used for GERMLINE, BEAGLE, DASH, and PLINK. Table S1. Alternative comparison of adjusted power on detecting pair-wise haplotype-sharing IBD between HaploShare, BEAGLE, GERMLINE, PLINK, DASH, and IBD-Groupon using an alternative method. Table S2. Ninety-five percent confidence intervals of the adjusted power in Table 1. Table S3. Comparison of adjusted power on detection of pair-wise haplotype-sharing IBD between HaploShare and others (sort by length). Table S4. Ninety-five percent confidence intervals of the adjusted power in Table 2. Table S5. Adjusted power of detection on shared extended haplotype significantly associated with diseases. Table S6. Adjusted power comparison on founder haplotypes generated by two different methods. Table S7. Adjusted power and rank of the founder haplotypes derived from sibpairs among all the regions found by HaploShare, BEAGLE fastIBD, IBD-Groupon, and DASH. Table S8. Comparison of adjusted power on detecting haplotype-sharing IBD between HaploShare, BEAGLE, GERMLINE, PLINK, DASH, and IBD-Groupon on simulated data from WTCCC data. Table S9. False positive rate and false discovery rate. Table S10. Power comparison with same false positive rate. Table S11. Ninety-five percent confidence intervals of the rank in Table 2. Table S12. A breakdown of time consumed by different steps by HaploShare. [file 13059_2015_662_MOESM1_ESM.doc]

**Additional file**

**HaploShare: Identification of shared extended haplotypes in patients associated with diseases**

Dingge Ying, Pak Chung Sham, David Keith Smith, Lu Zhang, Yu Lung Lau, and Wanling Yang


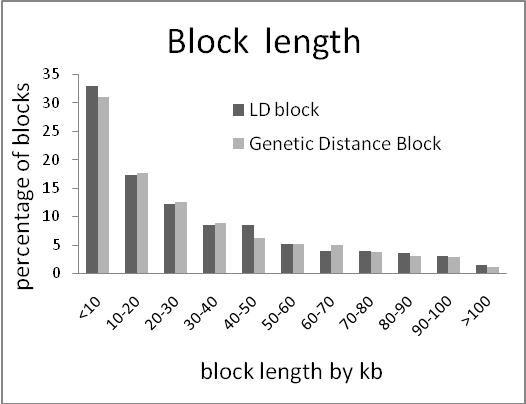

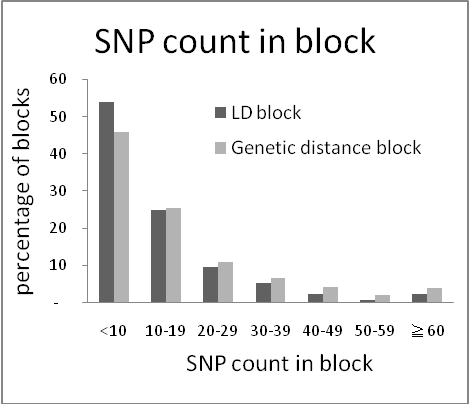


**Additional file 1: Figure S1. Number of SNPs and the length of the haplotype blocks.**

The figure shows the number of SNPs and length of the LD blocks based on the two LD block definitions when control data are provided (LD block, darker bars) or not (genetic distance block, lighter bars). LD blocks are defined as described in Methods. Both SNP count in blocks and block length showed similar frequency distribution with these two methods.

**Additional file 1: Figure S2. Estimation of false positive rate.** One thousand samples were randomly chosen in each simulation iteration and haplotype-sharing from the randomly chosen individuals without the process of simulating founder haplotypes was detected and evaluated using HaploShare. Sharing by 20 samples or fewer was evaluated for each simulation. The solid curve (both in the main figure and the inset) are the plots of the log_10_(likelihood ratio) from all the haplotype-sharing events surpassing the threshold for selecting pair-wise sharing of haplotypes for further analysis. The dashed curve (in both the main figure and the inset) is generated and smoothed from log_10_(likelihood ratio) of the best regions in each Monte-Carlo simulation iteration out of 1,000 repeats. The significance of the extended haplotypes shared is determined by the area under curve on the right tail of the dashed curve (0.05). The false positive rate is estimated as the area under the solid curve (shaded in the inset) to the right of the significance threshold (vertical dashed line in the inset) over the total area under the solid curve.


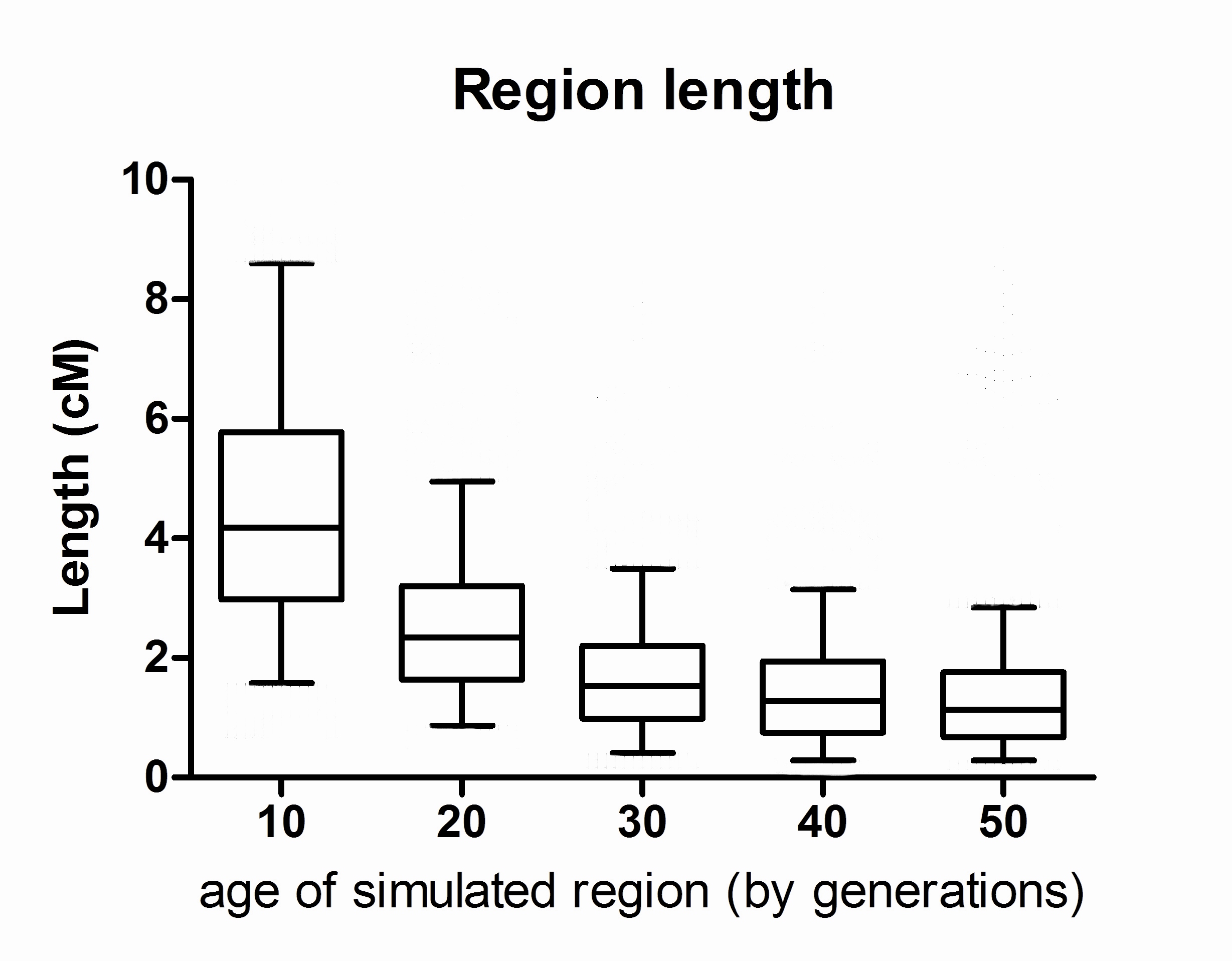


**Additional file 1: Figure S3.** **This graph summarizes the length of pair-wise sharing (cM, overlapped region only) of the simulated IBD regions of different age.** The top and the bottom bar are the 25th and 75th percentiles, and the sticker stand for 5th and 95th percentiles of the length of the shard haplotypes. The line in the bar indicates the median length.

**Quality control of the testing data**

The dataset contains 2,874 samples genotyped on 620,901 SNP markers using Illumina 610-Quad. About 30,000 SNPs failed missing data test (missing genotype >0.1), and 102,970 SNPs failed frequency test (MAF <0.005 ). And 1,965 SNPs failed Hardy-Weinberg equilibrium (*P* <0.0001). Seventeen individuals were removed for low genotyping rate (genotyping rate <0.9) and 51 individuals were removed for hidden relationship (20% genome-wide identity). After quality control, the remaining sample size was 2,805 samples and 514,105 SNPs.

**The parameters used for GERMLINE, BEAGLE, DASH, IBD-Groupon, and PLINK**

We ran GERMLINE 1.4.0 on phased data (generated by HaploShare) for IBD detection by adjusted parameters such as a sliding window size of 128 SNPs and a minimum length of IBD segments of 0.5cM (the default value was 5 cM). We also ran PLINK 1.07 on unphased data for IBD detection. We followed the basic settings of PLINK but changed the minimum length of IBD from 1,000 kb to 500 kb. SNPs with >1% missing genotypes and <5% minor allele frequency were removed, and pairwise LD based pruning was performed with a window size 100 SNPs, step size of 25 SNPs, and r^2^ threshold of 0.2. We ran Beagle 3.3.2 for both IBD mode and fastIBD mode and followed the default settings of the programs: prior IBD probability of 0.0001; IBD error rate of 0.005; IBD scale of 2.0 and iteration number of 10 for phasing.

For BEAGLE fastIBD, 10^-10^ was used as the threshold of fastIBD score (approximating the frequency of the shared haplotype) in both the adjusted power comparison and rank comparison, while 10^-12^ was also used for the rank comparison. When testing fast positive detection of fastIBD, randomly selected 1,000 samples were used to examine haplotypes shared pair-wise with fastIBD score <10^-10^ or 10^-12^, and the process was repeated for 100 times. Simulated founder haplotypes shared by different number of samples and mixed with the other samples were also used to test the detection by fastIBD (power) and their rank against the non-simulated haplotypes identified.

When comparing with PLINK for haplotype IBD associated with diseases, five out of 100 individuals were simulated to inherit a simulated common recent founder haplotype that is 10 to 50 generations of age. Another 500 individuals were used as controls, with the simulation process repeated 100 times. For PLINK, we used 500 kb and 100 SNPs as the selection criteria, and 10,000 permutation runs were performed for each simulation.

As suggested by DASH, pair-wise IBD results from GERMLINE was used as input for DASH. The window size was set as 500 kb and minimum cluster density was 0.6. The maximum r^2 value for identifying different haplotypes was set as 0.95. The minimum size of haplotype clusters was set as 3 so that DASH can report group IBDs shared by three samples or more.

As suggested in the paper, the output IBD file of BEAGLE fastIBD is used as input file for IBD-Groupon. The trunk file was also generated based on the BEAGLE fastIBD output file. The default value of 10E-8 was used as threshold.

| **Additional file 1: Table S1. Alternative comparison of adjusted power on detecting pair-wise haplotype-sharing IBD between HaploShare, BEAGLE, GERMLINE, PLINK, DASH, and IBD-Groupon using an alternative method*** | | | | | | | | | | | | | | | | | |
| --- | --- | --- | --- | --- | --- | --- | --- | --- | --- | --- | --- | --- | --- | --- | --- | --- | --- |
| Age of simulated regions (generations) | Length (cM) |  | HaploShare (multiple)* | |  | HaploShare (pair-wise)** | |  | BEAGLE IBD | BEAGLE fastIBD |  | PLINK |  | GERMLINE |  | DASH plus GERMLINE | BEAGLE plus IBD-Groupon |
|  |  | Threshold^#^ | 1 cM | 0.5 cM |  | 1 cM | 0.5 cM |  | N/A | N/A |  | 0.5 Mb |  | 0.5 cM |  | 0.5 cM | N/A |
| 50 | 1.14 |  | 26.8 | 30.4 |  | 9.5 | 13.5 |  | 10.5 | 2.3 |  | 0.0 |  | 17.9 |  | 15.2 | 5.6 |
| 40 | 1.28 |  | 33.3 | 36.9 |  | 15.8 | 21.9 |  | 13.9 | 12.9 |  | 0.0 |  | 18.6 |  | 17.1 | 15.8 |
| 30 | 1.53 |  | 92.5 | 93.0 |  | 48.9 | 71.5 |  | 22.3 | 21.7 |  | 0.0 |  | 22.3 |  | 24.5 | 26.6 |
| 20 | 2.35 |  | 96.1 | 96.4 |  | 64.8 | 82.6 |  | 52.8 | 54.0 |  | 3.5 |  | 31.3 |  | 37.2 | 56.2 |
| 10 | 4.18 |  | 100.0 | 100.0 |  | 89.3 | 93.9 |  | 94.2 | 92.4 |  | 31.2 |  | 40.2 |  | 48.2 | 93.4 |

*This alternative method only count the haplotype sharing when all the individuals with simulated haplotype are found. Missing one of the individuals would be counted as false detection, which is different from the method used in Table 1 (count as partial detection in Table 1).

| **Additional file 1: Table S2. Ninety-five percent confidence intervals of the adjusted power in Table 1.** | | | | | | | | | | | | | | | |
| --- | --- | --- | --- | --- | --- | --- | --- | --- | --- | --- | --- | --- | --- | --- | --- |
| Age of simulated regions (generations) | HaploShare (multiple)* | |  | HaploShare (pair-wise)** | |  | BEAGLE IBD | BEAGLE fastIBD |  | PLINK |  | GERMLINE |  | DASH plus GERMLINE | BEAGLE plus IBD-Groupon |
|  | 1 cM | 0.5 cM |  | 1 cM | 0.5 cM |  | N/A | N/A |  | 0.5 Mb |  | 0.5 cM |  | 0.5 cM | N/A |
| 50 | 42.2,48.0 | 44.7,50.9 |  | 28.2,32.2 | 36.2,41.2 |  | 34.2,39.0 | 28.8,32.8 |  | 0.00,0.00 |  | 39.2,44.6 |  | 34.0,38.7 | 27.3,31.1 |
| 40 | 48.8,55.0 | 51.6,57.6 |  | 33.6,38.2 | 43.9,49.9 |  | 38.7,44.1 | 38.1,43.5 |  | 0.00,0.00 |  | 41.8,47.6 |  | 37.6,42.9 | 36.7,41.8 |
| 30 | 94.5,95.1 | 94.8,95.4 |  | 64.4,68.8 | 80.2,82.6 |  | 46.0,52.4 | 45.6,52.0 |  | 17.9,20.3 |  | 46.0,52.4 |  | 44.6,50.8 | 46.0,52.4 |
| 20 | 97.6,97.8 | 97.7,97.9 |  | 78.7,81.3 | 89.5,90.7 |  | 71.5,74.9 | 72.2,75.6 |  | 36.7,41.8 |  | 58.6,63.6 |  | 59.8,64.7 | 71.9,75.4 |
| 10 | 100.0,100.0 | 100.0,100.0 |  | 94.2,95.0 | 96.7,97.1 |  | 96.9,97.3 | 96.0,96.4 |  | 63.4,67.8 |  | 68.1,72.0 |  | 70.2,74.3 | 96.2,96.7 |

| **Additional file 1: Table S3. Comparison of adjusted power on detection of pair-wise haplotype-sharing IBD between HaploShare and others (sort by length).** | | | | | | | | |
| --- | --- | --- | --- | --- | --- | --- | --- | --- |
|  | | | | | | | | |
| Length of simulated region (cM) | HaploShare | | HaploShare | | BEAGLE IBD | BEAGLE fastIBD | GERMLINE | PLINK |
|  | (Multiple) | | (Pair-wise) | |  |  |  |  |
|  | Min length cutoff=1cM | 0.5 cM | 1 cM | 0.5 cM | N/A | N/A | 0.5 cM | 0.5 Mb |
| <1 | 13.2 | 21.5 | 0.0 | 10.1 | 15.0 | 9.5 | 34.0 | 2.2 |
| 1-2 | 58.2 | 70.4 | 45.1 | 58.2 | 50.8 | 49.3 | 51.5 | 8.5 |
| 2-3 | 89.3 | 90.1 | 74.7 | 85.4 | 80.3 | 79.1 | 62.0 | 34.5 |
| 3-4 | 97.9 | 98.5 | 89.3 | 96.8 | 92.5 | 90.7 | 65.4 | 50.6 |
| 4-5 | 99.5 | 99.8 | 94.8 | 99.6 | 99.5 | 99.5 | 75.1 | 73.4 |
| >5 | 100.0 | 100.0 | 97.6 | 99.7 | 99.8 | 99.7 | 78.5 | 92.2 |

| **Additional file 1: Table S4. Ninety-five percent confidence intervals of the adjusted power in Table 2.** | | | | | | | | | | | | | | | | | | | | | | | | | | | | | | |
| --- | --- | --- | --- | --- | --- | --- | --- | --- | --- | --- | --- | --- | --- | --- | --- | --- | --- | --- | --- | --- | --- | --- | --- | --- | --- | --- | --- | --- | --- | --- |
| Software | |  | **HaploShare** | | | | | | | | | | | | |  | **BEAGLE fastIBD** | | | | | | |  | | **DASH+GERMLINE** | | | | |
| Threshold and total number of haplotypes found* | | |  | Pair-wise IBD >1.0 cM, *P* <0.05 5 found | | | | | |  | Pair-wise IBD >0.5 cM, *P* <0.05 32 found | | | | | |  | FastIBD score <10^-10^ 139 found |  | | <10^-12^  15 found | |  | | | Pair-wise IBD >0.5 cM 89 found | | | | |
| Number of samples sharing the simulated haplotype | | |  | 2 | | 5 | | 10 | |  | 2 | | 5 | | 10 | |  | 2 |  | | 2 | |  | | 2 | | | 5 | | 10 |
| Age of simulated founder haplotype (generations) | 10 |  | 71.8,76.2 | | 81.6,84.4 | | 98.9,99.1 | |  | 90.2,91.8 | | 94.6,95.4 | | 98.9,99.1 | |  | 94.6,95.4 | | |  | | 32.0,38.0 |  | | | 62.0,68.0 | 72.9,77.1 | | 80.5,83.5 | |
|  | 20 |  | 56.6,63.4 | | 76.1,79.9 | | 84.8,87.2 | |  | 70.7,75.3 | | 83.7,86.3 | | 89.2,90.9 | |  | 66.4,71.6 | | |  | | 19.2,22.8 |  | | | 49.0,57.0 | 62.0,68.0 | | 68.5,73.5 | |
|  | 30 |  | 31.1,36.9 | | 43.0,51.0 | | 56.6,63.4 | |  | 45.8,54.3 | | 60.9,67.1 | | 69.6,74.4 | |  | 37.5,44.5 | | |  | | 14.6,17.4 |  | | | 37.5,44.5 | 44.8,53.2 | | 47.9,56.1 | |
|  | 40 |  | 17.4,20.6 | | 23.8,28.2 | | 35.7,42.3 | |  | 35.7,42.3 | | 43.0,51.0 | | 49.0,57.0 | |  | 31.1,36.9 | | |  | | 11.0,13.0 |  | | | 32.0,38.0 | 35.7,42.3 | | 41.2,48.8 | |
|  | 50 |  | 8.2,9.8 | | 11.9,14.1 | | 21.0,25.0 | |  | 29.3,34.7 | | 35.7,42.3 | | 37.5,44.5 | |  | 25.6,30.4 | | |  | | 10.1,11.9 |  | | | 27.5,32.6 | 30.2,35.8 | | 33.9,40.1 | |

**Additional file 1: Table S5. Adjusted power of detection on shared extended haplotype significantly associated with diseases.**

| Age of simulated region (generations) | Median length  (5% - 95%) (cM) | Adjusted power by multiple individuals (%) | | | Adjusted power by pair-wise individuals (%) | | | PLINK | DASH + GERMLINE |
| --- | --- | --- | --- | --- | --- | --- | --- | --- | --- |
|  |  | Cutoff = 1 cM | 0.7 cM | 0.5 cM | 1 cM | 0.7 cM | 0.5 cM |  |  |
| 50 | 1.14 (0.29 - 2.84) | 22.9 | 27.1 | 33.2 | 10.5 | 17.9 | 22.0 | 0 | 22 |
| 40 | 1.28 (0.29 - 3.15) | 36.3 | 39.8 | 46.6 | 19.2 | 29.4 | 35.3 | 0 | 31 |
| 30 | 1.53 (0.41 - 3.50) | 58.2 | 62.7 | 65.5 | 37.5 | 50.1 | 54.1 | 3 | 45 |
| 20 | 2.35 (0.87 - 4.95) | 84.6 | 87.4 | 88.2 | 66.5 | 78.6 | 79.9 | 23 | 62 |
| 10 | 4.18 (1.58 - 8.60) | 99.5 | 99.4 | 99.7 | 93.2 | 95.3 | 96.1 | 48 | 76 |

**Additional file 1: Table S6. Adjusted power comparison on founder haplotypes generated by two different methods.**

| Founder haplotypes derived from sib-pairs | | Founder haplotypes from simulation | |
| --- | --- | --- | --- |
| Length (cM) | Power (%) | Length (cM) | Power (%) |
| 1 | 20.3 | < 1 | 8.9 |
| 2 | 60.8 | 1 - 2 | 53.2 |
| 3 | 91.6 | 2 - 3 | 88.7 |
| 4 | 98.1 | 3 - 4 | 97.3 |
| 5 | 98.7 | 4 - 5 | 99.3 |
|  |  | > 5 | 100.0 |

| **Additional file 1: Table S7. Adjusted power and rank of the founder haplotypes derived from sibpairs among all the regions found by HaploShare, BEAGLE fastIBD, IBD-Groupon and DASH.** Shown are the rank and (detection power%) of the simulated haplotypes. | | | | | | | | | | | | | | | | | | |
| --- | --- | --- | --- | --- | --- | --- | --- | --- | --- | --- | --- | --- | --- | --- | --- | --- | --- | --- |
| Software | |  | **HaploShare** | | | | | | |  | **BEAGLE fastIBD+IBD Groupon** | | | |  | **DASH+GERMLINE** | | |
| Threshold and total number of haplotypes found* | |  | Pair-wise IBD >1.0 cM, *P* <0.05 5 found | | |  | Pair-wise IBD >0.5 cM, *P* <0.05 32 found | | |  | FastIBD score <10^-10^ 181 found | | | |  | Pair-wise IBD >0.5 cM 89 found | | |
| Number of samples sharing the simulated haplotype | |  | 2 | 5 | 10 |  | 2 | 5 | 10 |  | 2 |  | 5 | 10 |  | 2 | 5 | 10 |
| Length of the simulated haplotype from sibpair | 5 cM |  | 1 (81) | 1 (89) | 1 (99) |  | 4 (95) | 3 (97) | 2 (99) |  | 30 (97) |  | 29 (97) | 29 (97) |  | 45 (68) | 38 (72) | 35 (75) |
|  | 4 cM |  | 1 (73) | 1 (79) | 1 (98) |  | 6 (90) | 5 (93) | 2 (99) |  | 35 (92) |  | 33 (93) | 31 (93) |  | 50 (65) | 48 (67) | 38 (71) |
|  | 3 cM |  | 1 (67) | 1 (75) | 1 (91) |  | 9 (81) | 7 (87) | 5 (94) |  | 39 (78) |  | 37 (80) | 35 (81) |  | 54 (58) | 52 (61) | 50 (64) |
|  | 2 cM |  | 2 (58) | 1 (71) | 1 (83) |  | 12 (69) | 11 (71) | 9 (83) |  | 43 (65) |  | 41 (68) | 40 (71) |  | 57 (51) | 58 (54) | 59 (55) |
|  | 1 cM |  | 5 (09) | 4 (11) | 3 (22) |  | 26 (30) | 27 (31) | 27 (31) |  | 118 (29) |  | 111 (32) | 108 (33) |  | 76 (30) | 77 (26) | 78 (27) |

| **Additional file 1: Table S8. Comparison of adjusted power on detecting haplotype-sharing IBD between HaploShare, BEAGLE, GERMLINE, PLINK, DASH, and IBD-Groupon on simulated data from WTCCC data.** | | | | | | | | | | | | | | | | | | |
| --- | --- | --- | --- | --- | --- | --- | --- | --- | --- | --- | --- | --- | --- | --- | --- | --- | --- | --- |
| Age of simulated regions (generations) | Length (cM) |  | HaploShare (Multiple) | |  | HaploShare (Pair-wise) | |  | BEAGLE IBD | BEAGLE fastIBD |  | PLINK |  | GERMLINE |  | DASH plus GERMLINE |  | BEAGLE plus IBD-Groupon |
|  |  | Threshold | 1 cM | 0.5 cM |  | 1 cM | 0.5 cM |  | N/A | N/A |  | 0.5 Mb |  | 0.5 cM |  | 0.5 cM |  | N/A |
| 50 | 1.14 |  | 41.2 | 44.3 |  | 18.5 | 28.4 |  | 26.4 | 18.9 |  | 0.0 |  | 34.5 |  | 30.5 |  | 22.1 |
| 40 | 1.28 |  | 48.1 | 62.1 |  | 28.4 | 39.3 |  | 32.8 | 31.5 |  | 0.0 |  | 37.7 |  | 38.9 |  | 34.8 |
| 30 | 1.53 |  | 97.5 | 93.5 |  | 63.8 | 77.6 |  | 44.1 | 41.7 |  | 6.8 |  | 43.0 |  | 47.9 |  | 48.7 |
| 20 | 2.35 |  | 98.7 | 97.9 |  | 80.1 | 90.2 |  | 67.3 | 68.5 |  | 31.9 |  | 57.7 |  | 60.1 |  | 75.0 |
| 10 | 4.18 |  | 100.0 | 100.0 |  | 95.2 | 98.7 |  | 97.1 | 94.9 |  | 65.2 |  | 68.1 |  | 74.6 |  | 95.7 |

**Additional file 1: Table S9. False positive rate and false discovery rate.**

| Pair-wise threshold | 1.0 cM | 0.5 cM |
| --- | --- | --- |
| Total number of regions | 7.33 x 10^5^ | 2.81 x 10^6^ |
| Number of regions reached significance (*P* <0.05) | 4.6 | 31.5 |
| False positive rate | 6.28 x 10^-6^ | 1.12 x 10^-5^ |
| False discovery rate  (assuming prior ratio of true vs. false = 0.0001) | 0.059 | 0.101 |
| False discovery rate  (assuming prior ratio of true vs. false = 0.001) | 0.006 | 0.011 |

| **Additional file 1: Table S10. Power comparison with same false positive rate.** | | | | | |
| --- | --- | --- | --- | --- | --- |
| Threshold for haplotype sharing | | 1 cM | | 0.5 cM | |
| Total haplotype sharing subject to test | | 7.33 x 10^5^ | | 2.81 x 10^6^ | |
| Program under test | | HaploShare | BEAGLE fastIBD  + IBD-Groupon | HaploShare | BEAGLE fastIBD  + IBD-Groupon |
| Program specific threshold | | Quasi *P* <0.161 | FastIBD score <10^-12^ | *P* <0.225 | FastIBD score <10^-10.3^ |
| Number of non-simulated haplotype sharing detected (false positive detections) | | 23 | 23 | 88 | 88 |
| **False positive rate** | | **3.14 x 10^-5^** | **3.14 x 10^-5^** | **3.14 x 10^-5^** | **3.14 x 10^-5^** |
| Number of individuals sharing the simulated haplotype | | 5 | | | |
|  |  | **Detection power** | | | |
| Age of simulated founder haplotype (generations) | 10 | **0.88** | **0.35** | **0.99** | **0.97** |
|  | 20 | **0.82** | **0.21** | **0.89** | **0.74** |
|  | 30 | **0.56** | **0.16** | **0.73** | **0.48** |
|  | 40 | **0.34** | **0.12** | **0.61** | **0.39** |
|  | 50 | **0.17** | **0.11** | **0.47** | **0.33** |

| **Additional file 1: Table S11. Ninety-five percent confidence intervals of the rank in Table 2.** | | | | | | | | | | | | | | | | | | | | | | | | | | | | | | |
| --- | --- | --- | --- | --- | --- | --- | --- | --- | --- | --- | --- | --- | --- | --- | --- | --- | --- | --- | --- | --- | --- | --- | --- | --- | --- | --- | --- | --- | --- | --- |
| Software | |  | **HaploShare** | | | | | | | | | | | | |  | **BEAGLE fastIBD** | | | | | | |  | | **DASH+GERMLINE** | | | | |
| Threshold and total number of haplotypes found* | | |  | Pair-wise IBD >1.0 cM, *P* <0.05 5 found | | | | | |  | Pair-wise IBD >0.5 cM, *P* <0.05 32 found | | | | | |  | FastIBD score <10^-10^ 139 found |  | | <10^-12^  15 found | |  | | | Pair-wise IBD >0.5 cM 89 found | | | | |
| Number of samples sharing the simulated haplotype | | |  | 2 | | 5 | | 10 | |  | 2 | | 5 | | 10 | |  | 2 |  | | 2 | |  | | 2 | | | 5 | | 10 |
| Age of simulated founder haplotype (generations) | 10 |  | 1.1,1.6 | | 1.0,1.5 | | 1.0,1.5 | |  | 3.3,4.7 | | 2.4,3.6 | | 2.2,3.2 | |  | 26.1,37.9 | | |  | | 9.0,13.0 |  | | | 40.8,59.3 | 37.5,54.5 | | 31.0,45.0 | |
|  | 20 |  | 1.2,1.7 | | 1.1,1.7 | | 1.0,1.5 | |  | 8.2,11.9 | | 6.5,9.5 | | 4.9,7.1 | |  | 36.7,53.3 | | |  | | 10.6,15.4 |  | | | 48.9,71.1 | 46.5,67.5 | | 44.0,64.0 | |
|  | 30 |  | 1.9,2.7 | | 1.7,2.5 | | 1.1,1.5 | |  | 16.3,23.7 | | 15.5,22.5 | | 12.2,17.8 | |  | 71.7,104.3 | | |  | | 11.4,16.6 |  | | | 55.4,80.6 | 53.0,77.0 | | 52.2,75.8 | |
|  | 40 |  | 2.8,4.0 | | 2.6,3.8 | | 2.0,2.8 | |  | 19.6,28.4 | | 18.7,27.3 | | 17.1,24.9 | |  | 87.2,126.8 | | |  | | 12.2,17.8 |  | | | 61.1,88.9 | 61.1,88.9 | | 61.1,88.9 | |
|  | 50 |  | 4.1,5.9 | | 3.3,4.7 | | 2.4,3.6 | |  | 22.0,32.0 | | 22.0,32.0 | | 22.0,32.0 | |  | 101.9,148.1 | | |  | | 12.2,17.8 |  | | | 61.9,90.1 | 65.2,94.8 | | 65.2,94.8 | |

**Additional file 1: Table S12.** A breakdown of time consumed by different steps by HaploShare.

| HaploShare analysis stage | | Computing time  (100 cases, 500 controls) | Computing time  (500 cases, 1,000 controls) |
| --- | --- | --- | --- |
| Catalog construction | | 8 h | 17 h |
| Phasing | | 2 min | 4 min |
| Case | Pair-wise-IBD | 5 min | 100 min |
|  | Group-IBD | 0.7 h | 18 h |
| Control | Pair-wise-IBD | 1.7 h | 9.3 h |
|  | Group-IBD | 18 h | 42 h |
| Total | | 28.5 h | 88 h |
